# Influence of Diet on the Bioaccessibility of Zn from Dietary Supplements: Findings from an In Vitro Digestion Model and Analytical Determinations

**DOI:** 10.3390/nu18010094

**Published:** 2025-12-27

**Authors:** Joanna Tokarczyk, Agnieszka Jaworowska, Dawid Kowalczyk, Monika Kasprzak, Paweł Jagielski, Wojciech Koch

**Affiliations:** 1 Department of Food and Nutrition, Medical University of Lublin, 4a Chodźki Str., 20-093 Lublin, Poland; joanna.tokarczyk@umlub.edu.pl (J.T.); agnieszka.jaworowska@umlub.edu.pl (A.J.); kowalczykdawid@outlook.com (D.K.); monika.kasprzak@umlub.edu.pl (M.K.); 2 Department of Nutrition and Drug Research, Institute of Public Health, Faculty of Health Sciences, Jagiellonian University Medical College, 31-066 Kraków, Poland; paweljan.jagielski@uj.edu.pl

**Keywords:** zinc, bioaccessibility, dietary supplements, diets, in vitro

## Abstract

Background: Zn is an essential mineral nutrient for human health. Its deficiency may result not only from insufficient intake but also from impaired absorption. Dietary components released from the food matrix during digestion can interact in ways that either enhance or inhibit mineral bioavailability. Objectives: The primary aim of this study was to evaluate the bioaccessibility of Zn from dietary supplements, particularly in the context of diet type, chemical form, and pharmaceutical formulation effects. Methods: The experiment was conducted using an in vitro gastrointestinal digestion model with cellulose dialysis membranes. Zn content after digestion was determined using flame atomic absorption spectrometry (F-AAS). The method employed had been previously developed and validated for use in determining the bioaccessibility of mineral nutrients. Results: The bioaccessibility of Zn from the standard, basic, and high-fiber diets was 19.43, 16.18, and 8.12%, respectively. In the presence of a standard diet, the bioaccessibility of Zn from dietary supplements was within the range 1.77–36.09%, in the presence of a basic diet, 1.05–35.86%; and in the presence of a high-fiber diet, 1.37–35.94%. The highest values were observed for zinc picolinate, whereas the lowest were determined for zinc oxide. Conclusions: A high-fiber diet significantly reduced Zn bioaccessibility. Bioaccessibility is also strongly dependent on the chemical form of zinc.

## 1. Introduction

Zn, the second most abundant trace element in the human body, is indispensable for maintaining normal physiological function. As the human organism lacks a significant zinc storage capacity, continuous dietary intake is required to sustain adequate levels [1]. Zinc serves as a structural and catalytic cofactor for more than 300 enzymes, thus participating in numerous biochemical processes [2], including intra- and intercellular signaling [3]. It plays a fundamental role in the regulation of cell differentiation, proliferation, and apoptosis [2,4,5]. This micronutrient is critical for the optimal function of the endocrine, reproductive, immune, and nervous systems. Disturbances in Zn homeostasis are associated with impaired reproductive functions as well as various neurodegenerative and psychiatric diseases [6,7,8,9].

Given the widespread prevalence of Zn deficiency and its significant health implications, maintaining adequate zinc status is of critical importance. Animal-derived foods, including oysters, meat, fish, eggs, milk, and dairy products, represent the richest dietary sources of zinc [10,11,12]. Plant-based foods such as nuts and legumes also provide appreciable amounts; however, they are consumed less frequently in most diets [13]. Cereal grains, which often constitute the dietary staple, typically contain zinc in the range of 23.13–33.76 mg/kg [14].Nevertheless, the absolute Zn content of a food item is less relevant to its nutritional contribution than its bioavailability. Zinc absorption is strongly influenced by dietary factors that either promote or inhibit intestinal uptake [15].

A balanced and diverse diet, incorporating a wide range of food groups, is considered the foundation of optimal human nutrition. However, the complexity of the food matrix introduces numerous interactions between dietary components, many of which are challenging to fully characterize [16]. Recent evidence indicates that compounds traditionally classified as antinutritional cannot be considered solely as such [17,18]. For example, dietary fiber and phytates, while recommended due to their established role in preventing various chronic diseases [19,20], can also inhibit the intestinal absorption of minerals, pharmaceuticals, and other bioactive compounds [21]. Conversely, a diverse diet also provides components that enhance the absorption of trace elements, including zinc. Proteins, peptides, and amino acids promote Zn absorption [15]. It is crucial to achieve the best possible understanding of the factors determining mineral bioavailability and, importantly, to translate this knowledge into practical applications.

This study used an in vitro approach assessing bioaccessibility rather than bioavailability, which are not interchangeable terms [15]. Bioavailability refers to the fraction of an ingested nutrient that is absorbed and utilized by the body, and its determination requires costly, labor-intensive, and ethically constrained in vivo studies [22,23,24,25]. Increasing attention has therefore been directed toward in vitro models that approximate in vivo conditions, including solubility and dialyzability assays, dynamic digestion systems (e.g., DIDGI, TIM), and Caco-2 cell models [26,27]. These methods offer lower cost, rapid and reproducible analysis, ease of application, and avoid ethical concerns, although they capture only the digestion and absorption phases [23,28]. Thus, the term bioaccessibility—defined as the fraction of a nutrient released from the food matrix and available for absorption—is used in this context [29]. The stimulated dialysis method applied during in vitro digestion is increasingly used to assess the bioaccessibility of minerals from both foods [30,31,32,33,34] and dietary supplements [16,27,35,36]. Dialysis methods have shown correlation with human mineral absorption studies [37]. However, it should be emphasized that Zn absorption efficiency from a single dose is higher than from daily, long-term supplementation. This effect is associated with a downregulation of zinc transporter expression in response to sustained high zinc intake [38]. In vitro methods are particularly useful for preliminary investigations, hypothesis generation, and screening, as well as for evaluating the effects of processing, preparation techniques, or specific food components on nutrient accessibility [27,28].

In parallel with growing nutritional awareness, interest in dietary supplementation has increased markedly. In 2017, approximately 70% of the Polish population reported the use of dietary supplements, and the global market is projected to reach approximately USD 300 billion by 2025. The COVID-19 pandemic further accelerated this trend, particularly for supplements containing zinc and vitamin D [39]. Zn supplements are available in various chemical forms, both organic and inorganic, which differ in physicochemical properties and, importantly, in bioavailability. From a consumer perspective, the selection of an appropriate form is critical to ensuring supplementation efficacy [15].

Current research on zinc bioavailability focuses mainly on the effects of zinc’s chemical form, specific foods, and particularly dietary phytate levels on absorption [15]. However, evidence regarding the impact of complete meals or whole diets remains limited. Such studies are essential for bridging the gap between nutrient analyses and real dietary conditions, allowing for a more accurate assessment of zinc absorption in mixed meals. The primary objective of this study was to evaluate how diet type influences the bioaccessibility of zinc from selected dietary supplements using an in vitro digestion model. Additionally, the study examined whether differences among zinc chemical forms are substantial and whether any form is less affected by dietary composition. Thus, the analysis focused on factors potentially shaping zinc absorption: its chemical form, pharmaceutical formulation, and diet type. To achieve this, zinc-containing dietary supplements from the Polish market were tested in combination with homogenized daily food rations composed of four meals. Bioaccessibility was assessed using a two-phase simulated gastrointestinal digestion procedure coupled with dialysis through a semipermeable cellulose membrane, followed by quantification via flame atomic absorption spectrometry (F-AAS).

## 2. Materials and Methods

### 2.1. Chemicals and Reagents

Pepsin (specific activity ≥ 500 U/mg), pancreatin (specific activity: 8xUSP), porcine bile extract, and dialysis tubing cellulose membranes, which were used during the stimulated digestion stage, were bought from Sigma-Aldrich (St. Louis, MO, USA). Sodium bicarbonate was purchased from Avantor Performance Materials (POCH, Gliwice, Poland). Sodium chloride was bought from Chempur (Piekary Śląskie, Poland). For the mineralization procedure and spectrometric determinations, nitric acid and hydrochloric acid of Suprapur Grade purchased from Merck (Darmstadt, Germany) were used. The standard solution of Zn was prepared from a stock solution of 1000 mg/L (Merck, Darmstadt, Germany). High-purity deionized water (resistivity of 18.2 MW cm) obtained using an Ultrapure Millipore Direct Q-R 3UV (Millipore, Bedford, MA, USA) was used during all determinations.

### 2.2. Materials

#### 2.2.1. Reconstructed Diet Duplicates

To estimate the bioaccessibility of Zn and evaluate the effects of three types of diets commonly consumed by healthy individuals, dietary models were developed based on the expertise of a professional dietitian (A.J.) and relevant scientific literature [40,41]. Three dietary patterns—standard, basic, and high-fiber—were reconstructed and applied in the experimental phase of this study. The nutritional composition of each diet was calculated using the Dieta 6.0 software (National Food and Nutrition Institute, Warsaw, Poland). The nutritional values of the diets are summarized in Table 1, while detailed qualitative and quantitative characteristics are provided in the Supplementary Material (Table S1).

Food products for the reconstruction of daily food rations (DFRs) were sourced from retail outlets in the Lublin region, with most items (meat, eggs, dairy, cereals, fruits, and vegetables) obtained from the local agricultural market. Duplicate diets were prepared using tap water from Lublin and seasoned with standard amounts of salt and spices; inedible parts (bones, peels, seeds) were removed. Diets were prepared according to validated laboratory procedures, regional culinary practices, and literature guidelines [16,35,42,43,44]. Only stainless steel utensils were used, and all equipment was cleaned with laboratory-grade detergent, then rinsed with dilute acid and deionized water to prevent cross-contamination. Prepared DFRs were weighed, homogenized with a titanium-blade homogenizer (Zelmer, Rzeszów, Poland), and stored at −20 °C until analysis.

#### 2.2.2. Dietary Supplements

In the present study, ten different dietary supplements containing Zn were selected. The Polish market offers a wide range of products containing this micronutrient. The selection of specific supplements was made based on the professional experience of the pharmacists participating in the study (J.T. and W.K.). Selection criteria included: regulatory status of the supplement, availability in the pharmaceutical market, consumer popularity, manufacturer reputation, and long shelf life. The selection also considered the diversity of Zn chemical forms. Full specifications of the analyzed supplements are presented in Table 2. Additionally, Table S2 in the Supplementary File provides a detailed composition of each dietary supplement. Each supplement was purchased from either a stationary or online pharmacy in three separate batches, and determinations were performed in triplicate for each batch.

### 2.3. The Gastrointestinal Enzymatic Model of In Vitro Digestion

In the present study, a two-phase enzymatic model of in vitro digestion was employed, encompassing both gastric and intestinal stages. In this study, the oral digestion phase was substituted with mechanical comminution of the food using a laboratory grinder. This approach is consistent with the INFOGEST protocol, which allows grinding as an alternative to mastication for solid foods. Minekus et al. also note that the oral phase may be omitted for liquid matrices. Although the protocol recommends a 2 min oral phase to enable salivary amylase activity, this duration exceeds the typical in vivo residence time. Shortening the in vitro oral phase, however, may compromise the accuracy and reproducibility of the digestion model [45]. Therefore, the authors elected to omit the oral phase entirely to avoid potential sources of variability that could influence the experimental outcomes. The omission of the oral phase was also related to the fact that supplements are swallowed and not digested in the oral cavity; therefore, including this phase in the study would cause a significant error compared to the actual conditions in which dietary supplements are consumed.

The procedure was conducted under physiologically relevant conditions, including the use of digestive enzymes at system-specific pH and temperature values that ensure enzymatic activity. The applied model is conceptually similar to the approach originally proposed by Miller et al. [46], but incorporates several modifications and improvements, such as the use of semi-permeable cellulose dialysis membranes and the simulation of gastrointestinal tract conditions. The procedure has been previously validated and demonstrated to provide reliable estimates of the bioavailability of mineral components [16,35] as well as other bioactive food compounds [47].

#### 2.3.1. Preparation of Dialysis Membranes

Dialysis membranes with a molecular weight cut-off (MWCO) of 14 kDa were used in the digestion procedure. Membranes were cut into 27 cm sections and preconditioned by soaking in 0.1 mol/L HCl for 12 h, followed by multiple rinses with ultrapure water. Prepared membranes were stored in deionized water until their use in the intestinal digestion stage.

#### 2.3.2. Two-Phase Model of In Vitro Digestion

Four experimental models were employed to simulate in vitro digestion. During the initial stage, corresponding to the gastric phase, the following samples were prepared:

- Enzymatic control sample: only 25 mL of ultrapure water (no diet; no dietary supplements).
- Diet sample: 25 g of homogenized diet to assess the bioaccessibility of the tested diet.
- Supplement-only sample: The manufacturer’s recommended portion of the dietary supplement mixed with 25 mL of ultrapure water, representing the bioaccessibility of the supplement under fasting conditions.
- Diet + supplement sample: The manufacturer’s recommended portion of the dietary supplement combined with 25 g of homogenized diet to evaluate the effect of the diet on Zn bioaccessibility from the dietary supplement.

For the P1 supplement, three tablets were used; for the P6 supplement, one-quarter of a tablet was used; and for the remaining products, one serving as recommended by the manufacturer was applied. During the experiment, zinc doses were selected in accordance with the manufacturer’s recommended intake because this strategy ensured practical relevance and reflected real-world patterns of consumer exposure.

All prepared samples were mixed with 25 mL of ultrapure water and 60 mg of NaCl. After adjusting the pH of the system to 2.0 using 2 mol/L HCl, a 10% pepsin solution was added (2 mL). Gastric digestion was carried out in tightly sealed bottles placed in a thermostatically controlled shaking water bath (37 °C) for 2 h.

The subsequent stage reproduced intestinal digestion and employed cellulose dialysis membranes. Before this step, the pH of the system was adjusted to 6.5 using 1 mol/L NaHCO_3_. The resulting fraction was treated with 5 mL of a 4% pancreatin solution with 2.5% *w*/*v* bile salts. Dialysis membranes containing the tested fraction, sealed with dedicated clips and placed in polypropylene laboratory containers containing 500 mL of ultrapure water, were shaken in a thermostatic water bath for 2 h at 37 °C.

**Figure 1:**
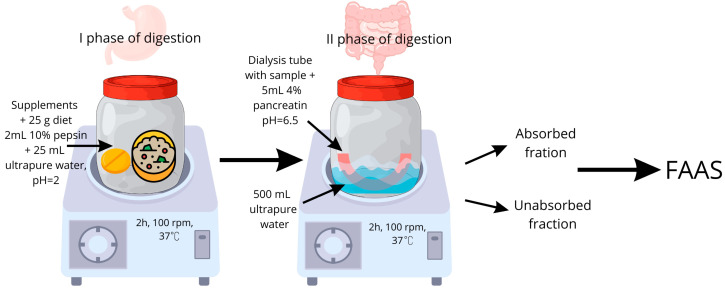
Schematic illustration of the applied procedure of the in vitro digestion.

Upon completion of the procedure, two fractions were obtained: the residue retained within the dialysis membranes and the dialysate, defined as the solution surrounding the membranes. Both fractions were subsequently subjected to analytical evaluation. A schematic representation of the experimental model is presented in Figure 1.

### 2.4. Mineralization Process

The digestion residues retained within the dialysis membranes were subjected to mineralization. Dried samples were placed in quartz crucibles and ashed in a muffle furnace with a temperature gradient up to 450 °C. After 72 h, mineralization was accelerated by the addition of 30% nitric acid (HNO_3_), which was evaporated, followed by re-heating of the samples at 300 °C. The resulting ash was treated with 15% hydrochloric acid (HCl), evaporated, and subsequently heated under cover for 5 min after the addition of another portion of 15% HCl solution. Obtained digests were quantitatively transferred into 25 mL test tubes, filtered through ashless filter paper to remove insoluble particles and silica, and filled up with ultrapure water.

### 2.5. Analytical Determination of Zn by the FAAS Method

The concentration of Zn was determined using atomic absorption spectrometry with an air-acetylene flame (FAAS) with deuterium background correction (λ = 213.9). The analysis was performed using iCE3000 (Thermo Scientific, Waltham, MA, USA). Instrumental settings are presented in Table S3 in the Supplementary Material. The equipment was calibrated using a standard solution of Zn (1000 mg/L) with appropriate dilutions (range 0.2–2.0 mg/L).

The method was checked and validated for accuracy and precision in the determination of trace elements by a simultaneous analysis of a mixture of flour and milk powder (7:3 *w*/*w*) with fortification of a known concentration of various elements, including Zn. The analysis conditions for the reference samples were the same as for the analytical samples. The reference samples were assayed in six replicates; the results are presented in Table 3.

Concentration of Zn in the dialysates obtained after in vitro digestion was determined directly using the F-AAS method. For this purpose, the dialysates were filtered using nylon syringe filters equipped with a 0.22 µm polyamide (PA) membrane (Bionovo, Legnica, Poland) and appropriately diluted with ultrapure water.

### 2.6. Calculation of the Bioaccessibility Value

Based on the results of the F-AAS determination of Zn content in dialysates and residue in dialysis tubes, the bioaccessibility of zinc was calculated, and expressed in percentage, by the formula below:

$$B\%=\frac{D}{D+T}\;\times \;100\%$$

where

*B*%—relative bioaccessibility of Zn;

*D*—amount of Zn (mg) in the dialysate;

*T*—amount of Zn (mg) in the digest of the dialysis tube residue.

*T* was calculated using the following equation:

$$T=\frac{\left(Ct-Ckt\right)\times \;Mp}{1000}$$

where

*Ct*—concentration of Zn in the digest of the dialysis tube residue (mg/L);

*Ckt*—concentration of Zn in the digest of the dialysis tube residue in the control sample (mg/L);

*Mp*—mass of digestive residue in the dialysis tube (g).

*D* was calculated using the following equation:

$$D=\frac{\left(Cd-Ckd\right)\;\times \;Vd}{1000}$$

where

*Cd*—concentration of Zn in the dialysate solution (mg/L);

*Ckd*—concentration of Zn in the control sample (mg/L);

*Vd*—volume of dialysate (mL).

### 2.7. Statistical Analysis

The results were reported as mean and standard deviation or median and first and third quartiles. The Shapiro–Wilk test was used to verify normal distribution for the analyzed variables. When the distribution of variables was normal, in order to examine the diversity of variance in the independent groups and to determine which differences between them were statistically significant, univariate analysis of variance (ANOVA) and, next, post hoc Tukey tests were used. If the distribution of variables deviated from the normal distribution, the Kruskal–Wallis test was used to check for differences between groups, and, next, post hoc pairwise comparisons All statistical analyses were performed using the statistical software packages PS IMAGO PRO (IBM SPSS Statistics 29). The value of *p* < 0.05 was considered significant. To lower the chance of type I errors, a Bonferroni correction for multiple comparisons was applied. 0.05 was divided by the number of tests, which is 325, resulting in a value of 0.0002. In this study, results were considered satisfactorily significant when the *p*-value was ≤0.0002.

## 3. Results

### 3.1. The Bioaccessibility of Zn from Dietary Supplements Under the Influence of Various Types of Diets

Table 4 summarizes the results of Zn bioaccessibility from dietary supplements under the influence of different diet types used in this study—standard, basic, and high-fiber—along with Zn bioaccessibility from the diets themselves. This table contains a statistical elaboration of results considering differences between various types of diets for particular dietary supplements. Moreover, Figure S1 presents an additional illustration of the obtained results with a detailed explanation of the interpretation of the provided letter and number symbols. Overall, Zn bioaccessibility varied significantly among the tested dietary models. The highest relative bioaccessibility was observed for the standard diet (19.43 ± 0.72%), while the lowest was noted for the high-fiber diet (8.12 ± 0.85%). The basic diet model yielded an intermediate value of 16.18 ± 0.20%. Significant differences were observed only between the high-fiber and standard diets (*p* ≤ 0.0002) and the high-fiber and basic diets (*p* ≤ 0.0002).

For the P1 (Zn gluconate) supplement, Zn bioaccessibility ranged from 3.63% to 7.54%. A significantly lower bioaccessibility was observed for the high-fiber diet compared to the basic (*p* ≤ 0.0002), standard (*p* ≤ 0.0002), and no-diet models (*p* ≤ 0.0002). No significant differences were found between the basic diet and the no-diet model, which represents the fasting intake model. The highest bioaccessibility (7.54%) was determined for the standard diet, which differed significantly from all other models (*p* ≤ 0.0002).

In the case of P2 supplement (Zn gluconate), the highest Zn bioaccessibility was observed in the presence of the standard diet (9.94%), although this value differed significantly from the high-fiber diet model. The basic diet model yielded a bioaccessibility of 8.01%, which differed significantly from high-fiber dietary model (*p* ≤ 0.0002). The lowest bioaccessibility (4.49%) was recorded for the high-fiber diet, significantly lower than in all other models (*p* ≤ 0.0002).

For P3 supplement, containing Zn gluconate, bioaccessibility values ranged from 5.40% to 16.53%. Zn bioaccessibility was significantly higher for the standard diet compared to both the basic and high-fiber diets (*p* ≤ 0.0002). No significant differences were observed between the basic and high-fiber diets. The lowest value (5.40%) was obtained in the no-diet model, which differed significantly from all diet models (*p* ≤ 0.0002).

In the case of P4 supplement (Zn gluconate), the highest bioaccessibility was observed in the no-diet model (13.92%), which differed significantly from all three diet models (*p* ≤ 0.0002). Among the diet models, bioaccessibility ranged from 6.10% to 9.08%, without significant differences between basic and high-fiber diets.

Similarly, for P5 supplement (Zn bisglycinate), the no-diet model exhibited the highest Zn bioaccessibility (19.35%), significantly higher than all diet models (*p* ≤ 0.0002). In the presence of diets, values ranged from 5.60% to 9.75%. The high-fiber diet resulted in significantly lower Zn bioaccessibility compared to the standard diet (*p* ≤ 0.0002), and the difference between the standard and basic diets was also significant (*p* ≤ 0.0002).

For P6 supplement, containing Zn lactate, the lowest bioaccessibility (3.17%) was recorded in the no-diet model, which was significantly different from all diet models (*p* ≤ 0.0002). Zn bioaccessibility under dietary conditions ranged from 6.53% to 8.74%. Significant differences were observed between the standard and basic diets (*p* ≤ 0.0002) and between the basic and high-fiber diets (*p* ≤ 0.0002).

In experiments with P7 supplement, Zn bioaccessibility ranged from 35.86% to 44.30%. Significantly higher values were observed in the no-diet model compared to all diet models (*p* ≤ 0.0002), while differences among diet models were not significant.

For P8 supplement (Zn citrate), Zn bioaccessibility ranged from 2.24% to 9.07%, with significant differences among models (*p* ≤ 0.0002). The lowest values were observed in the no-diet model, and the highest in the presence of the standard diet. In the model using diets, the lowest results were observed under the influence of a high-fiber diet (3.53%)

In the case of P9 supplement, containing Zn sulfate, Zn bioaccessibility values ranged from 2.00% to 8.75%. Significantly lower values were recorded in the no-diet model compared to all diet models (*p* ≤ 0.0002). No significant difference was observed between the standard and basic diets, whereas the highest and significantly different value was obtained under the high-fiber diet model (*p* ≤ 0.0002).

For P10 supplement (Zn oxide), average Zn bioaccessibility in the presence of diets ranged from 1.05% to 6.89%. In general, an average bioaccessibility of Zn in the presence of food matrix was low, and no differences between particular dietary models were determined. The highest bioaccessibility (6.89%) was found in the no-diet model, which differed significantly from all diet models (*p* ≤ 0.0002).

### 3.2. The Bioaccessibility of Zn from Dietary Supplements—Differences Between Particular Products

Table 4 presents the bioaccessibility of Zn from dietary supplements, taking into account the supplement type and, in particular, the chemical form of zinc present in each formulation. This table also presents statistical differences between particular dietary supplements. For fasting intake (model without diet), Zn bioaccessibility varied markedly across supplements, ranging from 2.00% for P9 (Zn sulfate) to 44.30% for P7 supplement. Among supplements containing zinc gluconate, bioaccessibility values ranged from 5.40% to 13.92%. No significant differences were found between P1 and P3. Second to P7 product, the highest Zn bioaccessibility was observed for P5 supplement (Zn bisglycinate) (19.35%), a significantly higher value than for most other products (*p* ≤ 0.0002). Bioaccessibility of 3.17% was determined for the P6 supplement (Zn lactate), which did not differ significantly from P8 (Zn citrate) and P9 (Zn sulfate) (2.00% and 2.24%, respectively). The P10 (Zn oxide) was characterized by the Zn bioaccessibility of 6.89%, which was not significantly different from P1 and P3 supplements, containing Zn gluconate.

When evaluating the standard diet model, the highest Zn bioaccessibility was again observed for P7 (36.09%), followed by a supplement containing Zn gluconate, P3 (16.53%). Only in the case of the P7 supplement was significantly higher bioaccessibility observed (*p* ≤ 0.0002) than from the standard diet alone (19.43%). Among gluconate-containing supplements, P4 did not differ significantly from P1 and P2. Similarly, no significant differences were found between supplements P1 and P2. For supplements containing zinc bisglycinate (P5), Zn citrate (P8), and Zn lactate (P6), bioaccessibility values were 9.75%, 9.07%, and 6.83%, respectively. Supplements containing Zn citrate and Zn bisglycinate did not differ significantly from those containing Zn gluconate, except in comparison to P3 supplement. The lowest bioaccessibility values were obtained for inorganic zinc forms—sulfate (P9, 4.30%) and oxide (P10, 1.77%). Both forms did not differ significantly in bioaccessibility values. No significant differences were also observed between supplement P9 (Zn sulfate) and supplements P1 (Zn gluconate) and P6 (Zn lactate).

In the model with the basic diet P7 (Zn picolinate) showed the highest Zn bioaccessibility (35.86%), significantly higher than all other supplements and the diet alone. Among zinc gluconate supplements, bioaccessibility ranged from 5.86% to 8.01%, with P1 differing significantly from P2 product (*p* ≤ 0.0002). For P5 (Zn bisglycinate), the value of 7.22% did not differ significantly from the gluconate-based, Zn sulfate and Zn lactate supplements. However, this value was significantly higher than those for supplements containing citrate and oxide forms. In this model, zinc citrate (P8, 5.02%) and zinc sulfate (P9, 5.30%) did not differ significantly, but both showed significantly higher bioaccessibility compared with zinc oxide (P10).

In the model simulating a high-fiber diet, the highest Zn bioaccessibility was again observed for P7, with statistically significant differences compared to all other supplements (*p* ≤ 0.0002). Among Zn gluconate-based supplements, Zn bioaccessibility ranged from 3.63% to 7.61%, with no significant differences between P1 and P2 supplements, P2 and P4, and also between P3 and P4. For P5 (Zn bisglycinate), bioaccessibility was 5.60%, without significant differences compared to Zn gluconate-based products. No significant differences were observed between the lactate and Zn bisglycinate forms and between lactate and gluconate-containing supplements, except for product P1. No significant differences were observed between the Zn bisglycinate form (P5) and Zn citrate (P8) in the model with a high-fiber diet. Notably, the P9 supplement (Zn sulfate) demonstrated significantly higher bioaccessibility under the influence of a high-fiber diet compared with supplements containing Zn gluconate, citrate, lactate, and bisglycinate forms. The lowest Zn bioaccessibility values, significantly lower than those of all other products, were observed for P10 (zinc oxide).

### 3.3. The Bioaccessibility of Zn from Dietary Supplements—Impact of Pharmaceutical Formula

An additional objective of this study was to assess the effect of pharmaceutical formulation on the bioaccessibility of Zn from dietary supplements. The corresponding results are presented in Table 5. Most of the tested supplements were available in tablet form, with one supplement formulated as a coated tablet and two in capsule form. Across all dietary models, the highest Zn bioaccessibility was observed for the capsule formulations. However, significant differences between capsules and tablets were found only in the model simulating a high-fiber (high-fiber) diet (*p* ≤ 0.0002). Significant differences were also noted between tablets and coated tablets under both the standard and high-fiber dietary conditions. Notably, tablet formulations exhibited significantly lower Zn bioaccessibility under the high-fiber diet compared to the other dietary models (*p* ≤ 0.0002). For coated tablets, the median Zn bioaccessibility under the standard diet was 16.5%. Significant differences were detected between this diet and two dietary models: without diet and basic diet (*p* ≤ 0.0002) No significant differences were observed among the dietary models for capsule formulations, indicating a consistent Zn bioaccessibility profile across dietary conditions.

## 4. Discussion

Zn released from the food matrix in its ionized form is primarily absorbed in the duodenum and jejunum. The efficiency of absorption is dependent on dietary intake, while systemic zinc homeostasis is partially maintained through the modulation of intestinal absorption and the regulation of urinary and fecal excretion. However, these adaptive mechanisms are not always sufficient to prevent zinc deficiency [48,49]. Importantly, Zn bioavailability is determined not only by physiological processes but also by dietary factors. Digestion of the food matrix releases ligands that can enter into complex, often elusive, interactions with other dietary components [50,51]. The principal mechanisms include the binding of zinc ions by phytates with the formation of insoluble complexes, competition with iron for intestinal transporters, and zinc complexation with amino acids and peptides [51]. Moreover, in the context of supplementation, the chemical form of Zn, along with its physicochemical properties—including stability, water solubility, redox potential, and sensitivity to environmental pH—play a decisive role in determining its bioavailability [52].

The bioavailability of Zn has been investigated for many years. According to Maares et al., absorption from a normal, varied diet ranges between 16% and 50% [51]. Hunt et al. further reported that zinc is absorbed more efficiently from non-vegetarian diets (33%) compared to lacto-ovo-vegetarian diets (26%) [53]. In the present study, the bioaccessibility of dietary Zn, representing three types of normal, mixed models of diets, was determined within the range of 8.12–19.43%, depending on the type of diet. Notably, diets with a high fiber content were associated with reduced Zn bioaccessibility. In this case results of in vitro determinations performed in the current study are in agreement with in vivo bioavailability studies in which the inhibition of Zn by the components of dietary fiber was revealed [21]. Consistently, Foster et al. demonstrated that high-fiber diets are correlated with an increased risk of Zn deficiency [54].

The reduced absorption of Zn in high-fiber diets is primarily attributed to the presence of phytic acid and its derivatives, which form insoluble complexes with minerals that are poorly available for absorption. Latunde-Dada et al. and Mayer-Labba et al. demonstrated that the bioavailability of Zn from plant-based meat substitutes is significantly lower compared to meat products [55,56]. Importantly, not only the presence of phytates but also the phytate-to-zinc molar ratio determines zinc absorption efficiency. Zn absorption has been shown to decrease from approximately 21 to 11–16% at molar ratios of 5–15, and to less than 11% at ratios exceeding 15 [57]. In recognition of the inhibitory effect of phytates on Zn bioavailability, both EFSA and WHO have proposed differentiated dietary zinc intake recommendations, stratified according to dietary phytate content [58,59].

Phytates are hydrolyzed by phytases, which are present in limited amounts in the human intestine. Their degradation occurs primarily through the activity of plant- and microbially derived phytases. Plant phytases are activated during fermentation and heat treatment of foods [51]. In the present study, the high-fiber diet was largely composed of non-fermented and non-heat-treated products, with bran constituting a major component. In such products, phytase activity is low. Nevertheless, high-fiber diets are also known to promote the growth of beneficial intestinal microbiota capable of phytase production [60,61,62]. Particularly in the context of this type of diet, the in vitro dialysis model of digestion may underestimate the bioaccessibility of nutrients. Yin et al., employing an in vitro digestion model combined with the Simulator of the Human Intestinal Microbial Ecosystem (SHIME), demonstrated that neglecting the colonic phase can result in the underestimation of the bioaccessibility of zinc, as well as iron and manganese [63].

The current study demonstrated a correlation between high dietary fiber intake and reduced Zn bioaccessibility, both from whole diets and from organic forms of zinc in the studied dietary supplements. The reduction in bioaccessibility may be influenced not only by fiber itself but also by the elevated Ca content in the high-fiber diet model employed. However, the role of Ca in this interaction remains inconclusive. In vivo studies have shown that Ca, in the presence of phytates, can negatively affect Zn bioavailability [64,65]. Conversely, Miller et al. reported a small but opposite effect of Ca under similar conditions [66], while Hunt et al. observed an inhibitory effect of phytates but no significant influence of Ca [67].

Diets lower in fiber—classified as basic and standard—contain higher amounts of highly bioavailable Zn of animal origin, compared to high-fiber diets. However, in the case of the Zn sulfate supplement, the highest bioaccessibility was observed in the presence of a high-fiber diet. This finding is unexpected, as previous studies in rats conducted by Lo et al. demonstrated that Zn bioavailability was significantly reduced in animals consuming phytate-rich diets [68]. Similarly, for the zinc oxide supplement, no significant differences in zinc bioaccessibility were observed among the diets. These supplements also contained an additional mineral component, vitamins, and polyphenol-rich plant extracts, including *Equisetum arvense* L. herb, *Urtica dioica* herb, bamboo shoots, and *Panax ginseng* root extracts. Previous studies by Sreenivasulu et al. investigated the effects of polyphenol-rich beverages—such as red wine, red grape juice, and green tea—on Zn uptake in Caco-2 cells, demonstrating enhanced Zn uptake and metallothionein expression [69]. In contrast, Brnić et al. reported an inhibitory effect of sorghum polyphenols on Zn absorption, but only in the presence of phytates, with no inhibition observed when phytates were absent [70]. Furthermore, Kim et al. found that grape seed extract reduced Zn absorption in Caco-2 cells, whereas epigallocatechin-3-gallate (EGCG) and green tea extract showed no such effect. The inhibitory action of grape seed extract has been attributed to zinc chelation by proanthocyanidins [71].

When Zn was supplemented in the forms of gluconate, bisglycinate, and citrate, the standard diet was associated with higher Zn bioaccessibility in comparison to the basic diet. Proteins, particularly those of animal origin, along with their degradation products (amino acids and peptides), are known to enhance zinc absorption [51,72]. Interestingly, the standard diet in this study contained less protein, including animal protein, than the basic diet, suggesting that the critical factor is fiber content, which was lower in the standard diet. Additionally, the higher Ca content in the basic diet (943 mg), combined with phytates, likely further reduced Zn absorption. The lowest calcium content was observed in the standard diet (612.6 mg), whereas the highest was found in the high-fiber diet (1625.4 mg). Another nutrient that may modulate zinc bioaccessibility in different dietary contexts is vitamin C. The vitamin C content in the standard, high-fiber, and basic diets was 273.6, 146.4, and 136.8 mg, respectively. Previous research by Zhang et al. demonstrated that vitamin C enhances Zn bioaccessibility from rice. In contrast, vitamin E has been reported to reduce zinc absorption [73]. Consistent with these findings, the present study revealed higher zinc bioaccessibility from the standard diet, which contained markedly higher levels of vitamin C compared to the basic and high-fiber diets, and lower levels of vitamin E (15.2 mg) compared to the high-fiber diet (100 mg). The vitamin E content between the basic and standard diets was not significant (14.9 mg and 15.2 mg, respectively).

Notably, for Zn picolinate, no significant differences in bioaccessibility were observed among the various diet types used in the current study. Zn picolinate is often regarded as one of the most bioavailable forms of this element. Bawiec et al. demonstrated that picolinate also showed the highest bioavailability among various chromium compounds [16]. Similarly, Barrie et al. reported that, of the three zinc forms—glycinate, citrate, and picolinate—administered for four weeks to 15 healthy volunteers, only picolinate significantly increased zinc levels in hair, urine, and red blood cells [74]. In contrast, findings from DiSilvestro et al. indicated that among glycinate, gluconate, picolinate, and oxide, glycinate demonstrated the best absorption. Plasma Zn levels following picolinate supplementation were comparable to those observed with zinc oxide. However, based on red blood cell zinc content (area under the curve and rank scores), the order of bioavailability was glycinate > picolinate > oxide > gluconate [75]. More recent studies by Ośko et al. reported that Zn bisglycinate and gluconate exhibited greater bioaccessibility than picolinate, though dietary effects were not assessed [27]. Notably, the present study confirms that the bioaccessibility of Zn picolinate is not influenced by diet composition. This form is of particular interest, as picolinic acid is a natural metabolite of tryptophan that plays an essential role in zinc transport and has additionally been shown to possess antiviral properties [76].

According to Shkembi, Zn absorption from food supplements reaches 60–70% when ingested on an empty stomach or with a liquid meal [54]. These findings are not fully consistent with the results of the present study. None of the supplements evaluated in the present study demonstrated bioaccessibility values as high as those previously reported, which is in agreement with recent findings of Ośko et al. [27]. Indeed, supplements such as P4 (zinc gluconate), P5 (zinc bisglycinate), P7 (zinc picolinate), and P10 (zinc oxide) exhibited higher bioaccessibility under fasting conditions than when administered with diets. In contrast, the remaining products were less efficiently absorbed in the fasted state. Improved bioaccessibility in the presence of diet for products containing gluconate, citrate, lactate, or sulfate is an additional advantage, as these salts may cause gastrointestinal adverse effects, including nausea and vomiting, when consumed on an empty stomach [77].

Previous research has generally indicated that Zn is more bioavailable from organic compounds than from inorganic ones, a trend largely confirmed in this study. An exception was observed for zinc oxide, which demonstrated superior bioaccessibility in the fasting model compared with citrate, lactate, and sulfate. This effect may be attributed to the relatively high solubility of zinc oxide in dilute acids and bases [52,78]. ZnO exhibits its highest solubility at pH 2–4, which corresponds to typical gastric conditions [79]. Avramescu et al. reported that the solubility of bulk ZnO at pH 7 was only 0.95%, whereas at pH 1.5 it increased to 88.5% [80]. The bioavailability of ZnO is influenced, among other factors, by its dissolution kinetics. The dissolution rate can modulate interactions with food components, affect intestinal absorption, and determine the transit time of the compound through the gastrointestinal tract. Importantly, the time required to reach dissolution equilibrium often exceeds the duration of the digestive process itself. Cardoso et al. also emphasized that dissolution should not occur too rapidly, as an excessive release of Zn^2+^ ions in acidic conditions may promote their binding to phytates, which subsequently precipitate in the duodenum at pH values close to 7 (approximately pH 6) [81]. However, in individuals with altered gastric pH—for example, due to the use of proton pump inhibitors or antacids—solubility and, consequently, bioavailability are markedly reduced [52]. It should be noted that solubility does not necessarily translate to dialyzability. Guillem et al. reported that zinc oxide demonstrated higher dialyzability from milk formulas compared with lactate, citrate, or sulfate [82]. In the dietary models applied in the present study, however, zinc sulfate was more bioavailable than zinc oxide. Wolfe et al., in a study involving 48 pregnant women, also observed higher plasma Zn concentrations in the group supplemented with 20 mg of zinc sulfate compared with the group receiving 25 mg of zinc oxide [83]. Conversely, Rosado et al. reported no significant differences in zinc absorption between sulfate and oxide [84], a finding supported by subsequent studies by Hotz et al. and Herman et al. [85,86]. Similarly, De Romaña, who investigated Zn fortification of low-phytate bread and high-phytate oatmeal with either zinc oxide or sulfate, found no significant interaction between diet type and fortification, although a slight advantage of sulfate in zinc absorption was observed [87]. Interestingly, in the present study, zinc sulfate exhibited higher bioaccessibility than the organic forms—gluconate, bisglycinate, lactate, and citrate—when administered with the high-fiber diet. This finding contrasts with previous results reported by Schlegel et al., who observed higher Zn bioavailability from zinc glycinate than from zinc sulfate in rats fed a semisynthetic basal diet supplemented with pure sodium phytate (8 g/kg) [88]. The unusual result observed should be interpreted with caution and underscores the need for further research to clarify the underlying interactions. In the case of zinc sulfate, factors beyond the chemical form and diet—such as other components of the preparation with potential buffering properties—may have influenced the system’s pH and, consequently, the solubility of the salt.

Apart from zinc sulfate, the most frequently used form of Zn supplementation is zinc gluconate. In the present study, the relative bioaccessibility of gluconate-containing supplements ranged from 5.40 to 13.92% in the fasted state, and from 3.63 to 16.53% in diet-based models. Notably, considerable variability was observed among preparations containing the same Zn gluconate salt. Although the authors did not conduct a qualitative evaluation of individual products, it is essential to note that, under Polish regulations, dietary supplements are not subject to mandatory quality testing before market authorization. Therefore, the observed discrepancies in bioaccessibility may be attributed to inconsistencies between the manufacturers’ declared composition and the actual content of the preparations. Sapota et al. reported that Zn gluconate exhibited higher bioavailability in the rat prostate compared to zinc sulfate or zinc citrate. According to the authors, this form of supplementation may be particularly beneficial for men, given that zinc intake has been associated with a reduced risk of prostate cancer, partly through protection against damage caused by reactive oxygen species [89]. Additionally, Del Rio et al. highlighted the beneficial effects of Zn gluconate supplementation in promoting the restoration of the intestinal epithelial barrier, thereby reducing transmucosal permeability associated with inflammatory bowel disease, celiac disease, autism spectrum disorders, and Alzheimer’s disease [90]. In contrast, under fasting conditions, Zn bisglycinate demonstrated higher bioaccessibility than zinc gluconate, a finding consistent with those of Ośko et al. [27]. In diet-based models, however, the relative bioaccessibility values of gluconate- and bisglycinate-containing supplements did not differ significantly. An exception was observed for P3 supplement (zinc gluconate), where the determined bioaccessibility in the presence of a standard diet reached 16.53%. However, further experimental studies including a greater number of products containing zinc bisglycinate are required to confirm this observation. Previous clinical studies conducted independently by DiSilvestro et al. and Gandia et al. confirmed the improved bioavailability of zinc when combined with bisglycinate [91,92]. Gandia et al. attributed the enhanced absorption of Zn from bisglycinate to its distinct chemical properties, particularly its molecular structure. The presence of two functional groups enables the formation of both covalent and coordination bonds, facilitating the creation of a sterically and energetically stable ring structure with Zn. In addition, the compound’s relatively low molecular weight (<1000 Daltons) and its stability within a pH range of 2–6 appear to confer protection against interactions with dietary components and concomitantly administered medications [92]. Wegmüller et al. evaluated the absorption fraction of Zn from three forms: gluconate, citrate, and oxide. In this study, fifteen healthy adults received the supplements under fasting conditions. Zinc absorption was comparable between citrate and gluconate, but significantly lower for oxide [93]. In contrast, the present study demonstrated higher bioaccessibility for gluconate than for citrate under fasting conditions, which is consistent with the findings reported by Ośko et al. [27]. For the standard diet model, the results of bioaccessibility of Zn from supplements containing gluconate and citrate were similar, except for the P3 supplement. On the other hand, better absorption from supplements containing gluconate was observed under the influence of the basic diet. However, in the presence of a high-fiber diet, significantly higher bioaccessibility results were observed for the two preparations containing gluconate compared to the product with Zn citrate.

Capsule-based dietary supplements were observed to demonstrate higher bioaccessibility compared to tablets and coated tablets. However, this observation should be considered preliminary, as the study encompassed only a limited range of pharmaceutical forms. Furthermore, the capsule formulations analyzed included two chemical forms of zinc—picolinate and sulfate—with markedly different bioaccessibility profiles. This finding suggests that the chemical form of Zn may have a stronger impact on bioaccessibility than the dosage form itself. Nevertheless, as shown by Ośko et al., in the case of zinc gluconate preparations, encapsulated formulations achieved higher bioaccessibility than tablet-based products [27]. In a controlled study by Johnson et al., involving 10 healthy men and 5 healthy women, the bioavailability of key constituents, including zinc from zinc oxide, from a dietary supplement administered in gelatin capsules and tablets was evaluated. The investigation revealed a distinct bimodal absorption profile, characterized by a transient reduction in serum Zn concentrations between two phases of increased uptake. The estimated bioavailability of zinc ranged from 7% to 11%. No significant differences were observed between capsule and tablet formulations when the complete supplement dose (69.6 mg ZnO) was administered [94].

When interpreting the data obtained in this study, several limitations should be taken into account, including those related to the number of dietary supplements used. Although the most commonly available and frequently consumed forms of Zn were selected, the market for dietary supplements is, in fact, extensive. Moreover, the ease of marketing authorization and the continuously growing demand contribute to the constant emergence of new products, both in the pharmacy and non-pharmacy sectors. Another limitation concerns the use of complete food rations, which consist of four meals. In real-life conditions, dietary supplements are consumed at various times of day, either on an empty stomach or in combination with different types of meals. Nevertheless, the application of dietary homogenates in this study made it possible to assess the impact of the most common dietary patterns among adults on zinc bioaccessibility.

The final results also largely depend on the method applied to assess bioaccessibility. In vitro digestion methods are among the most commonly used, as evidenced by the development of the INFOGEST protocol, which is a standard in research using in vitro digestion models. Although this protocol was developed to best reflect physiological conditions, it is acknowledged that it has its limitations. First and foremost, dialysis membranes reflect only passive transport, which occurs physiologically at high levels of Zn intake. By contrast, active transport, mediated by two classes of transporters (ZnT and ZIP proteins), predominates under typical conditions [95]. Additionally, bioavailability results for amino acid complexes with zinc (e.g., glycinate) may be underestimated because, as Sauer et al. have shown, such complexes can be transported with the participation of amino acid transporters [72]. Gomez et al. demonstrated in their study on Zn absorption from milk formulas that, despite the use of membranes with pore sizes of 10–12 kDa, zinc bound to molecules of 1–7 kDa was not fully dialyzed [34]. Another issue relates to the underestimation of zinc bioavailability resulting from the omission of processes occurring in the large intestine [63]. Finally, maintaining physiologically relevant pH conditions during digestion simulation is also crucial. In the applied model, the gastric digestion phase was conducted at pH 2, which reflects the typical conditions of the adult stomach. In children—one of the populations at increased risk of Zn deficiency—the gastric pH is usually closer to 4 [96]. Johnston et al. demonstrated that increasing the pH from 2 to 4 reduces pepsin activity by approximately 40%, which consequently decreases the absorption of both Zn and Fe [97]. Similarly, Kruger et al. reported lower Zn absorption at pH 4 compared with pH 2 [96]. Multicomponent dietary supplements may exhibit pH-buffering properties. Moreover, individuals using antacids should consider appropriate adjustments to zinc supplementation, as elevated gastric pH can impair zinc absorption. It should be noted that the present study did not include analyses to verify whether the zinc content in the tested supplements was consistent with the manufacturers’ declarations. In addition, speciation analysis was not performed. Future research should consider verifying the manufacturers’ claims regarding the chemical forms of zinc present in dietary supplements. Moreover, the level of Zn content in tablets or capsules may influence the bioaccessibility parameter, which applies to both in vitro and in vivo models. However, the influence of Zn content in a given pharmaceutical form on the bioaccessibility values was not investigated in these experiments.

The findings of this experiment have practical implications and may be of value to physicians, pharmacists, and dietitians when advising patients requiring balanced supplementation. They can inform evidence-based guidelines for selecting supplements that are effective, safe, and independent of dietary habits, thereby supporting clinicians and dietitians in personalizing supplementation. For manufacturers, the findings provide a basis for optimizing supplement formulations, improving bioaccessibility, and minimizing potential interactions with food. Additionally, the data can be used to design educational materials for consumers, highlighting proper use and safety considerations. Finally, these results could serve as a reference for further research, including studies on supplement-food interactions and testing new formulations in dynamic in vitro or clinical models, which are more expensive but better suited to assessing bioaccessibility/bioavailability. As previously indicated, the current study therefore provides a good screening tool. It is also worth noting that recent research on zinc bioavailability has increasingly focused on evaluating the effects of combining zinc with bioactive peptides [98,99], amino acid complexes, and polysaccharide complexes [100,101]. These compounds have demonstrated considerable potential to enhance zinc absorption and improve the efficacy of zinc supplementation [15].

## 5. Conclusions

The present study highlights the significant impact of the complex food matrix on Zn bioaccessibility, particularly in foods rich in dietary fiber and phytates. Moreover, the presence of animal-derived proteins plays a crucial role in enhancing Zn absorption. Individuals adhering primarily to plant-based diets, and thereby excluding animal protein sources, are at an increased risk of Zn deficiency. For these populations, appropriate zinc supplementation strategies are essential, while still maintaining adequate dietary fiber intake due to its numerous health benefits. Results of the current study indicate that zinc picolinate exhibits superior bioaccessibility and reduced interaction with food components compared with other chemical forms of zinc. Although zinc picolinate is relatively more expensive than zinc sulfate, economic considerations are particularly relevant in developing countries, where diets are predominantly cereal-based. In such populations, Zn sulfate may represent a cost-effective and efficient option for addressing zinc deficiency, as our results suggest improved bioaccessibility of this form compared to other zinc salts, with the exception of picolinate, under high-fiber dietary conditions. Nevertheless, these findings warrant further confirmation. In vitro digestion models employing semipermeable dialysis membranes may serve as a valuable tool for such investigations. When combined with analytical and statistical approaches, this model provides practical insights for clinicians, consumers, and manufacturers of dietary supplements. Furthermore, the method can be readily adapted to evaluate the effects of various dietary and environmental factors on the bioaccessibility of mineral components.

## Figures and Tables

**Table 1:** Selected nutritional parameters of diets used in the study.

Parameter		Diet		
		Basic	Standard	High-Fiber
Energy (kcal)		2268	2035	2130
Total weight (g)		2470	2705	2845
Proteins (g)		109.3 (19.3% of E *)	93.8 (18.7% of E)	94.6 (17.9% of E)
Fats (g)		94.2 (36.7% of E)	63.4 (27.5% of E)	74.3 (30.8% of E)
Carbohydrates (g)		286.2 (44% of E)	302.4 (53.8% of E)	328 (51.3% of E)
Fiber (g)		41.2	32.7	60
Vitamins	A (µg)	586.4	1677.2	705.4
	C (mg)	136.8	273.6	146.4
	E (mg)	14.9	15.2	100
Mineral components (mg)	Na	2900	2689.7	2194.4
	K	4360	4940	4645.7
	Ca	943	612.6	1625.4
	Mg	514.4	487.2	767.1
	Fe	16.9	15	21.7
	Zn	17.9	10.6	20

* Percentage of energy.

**Table 2:** Full specification of supplements containing Zn used in the study.

Dietary Supplement	Chemical Form	Zn Content Declared by Producer [mg]	Pharmaceutical Form	Supplement Type
P1	zinc gluconate	5.3	tablet	mineral
P2	zinc gluconate	15	tablet	single mineral
P3	zinc gluconate	10	coated tablet	single mineral
P4	zinc gluconate	15	tablet	single mineral
P5	zinc bisglicynate	10	tablet	vitamin-mineral with plant extracts
P6	zinc lactate	15	tablet	single mineral
P7	zinc picolinate	22	capsule	single mineral
P8	zinc citrate	15	tablet	vitamin-mineral
P9	zinc sulfate	2.5	capsule	vitamin-mineral with plant extracts
P10	zinc oxide	10	tablet	vitamin-mineral with plant extract

**Table 3:** Concentration of Zn in the reference material.

Parameter	Zn
Theoretical value (mg/kg)	24
Determined value (mg/kg)	25.7
	25.8
	22.7
	24.3
	22.9
	24.3
Average	24.3
SD	1.21
RSD (%)	4.98
Recovery (%)	101.0
LOD (µg/kg)	41
LOQ (µg/kg)	152

SD—Standard Deviation; RSD—Relative Standard Deviation; LOD—Limit of Detection; LOQ—Limit of Quantification.

**Table 4:** Bioaccessibility [%] of Zinc from dietary supplements under the influence of various types of diets. The means sharing the same letter in a row show a statistically significant difference between diets at *p* ≤ 0.0002. Considering differences between particular products for specific types of diets, the means sharing the same number in a column show a statistically significant difference between supplements at *p* ≤ 0.0002 *.

Dietary Supplement	Chemical Form	Without Diet ^a^ (n = 9) X ± SD	Standard Diet ^b^ (n = 9) X ± SD	Basic Diet ^c^ (n = 9) X ± SD	High-Fiber Diet ^d^ (n = 9) X ± SD	ANOVA *p*
Without ^(0)^	-	-	19.43 ± 0.72 ^d (1,2,4,5,6,7,8,9,10)^	16.18 ± 0.20 ^d (1,2,3,4,5,6,7,8,9,10)^	8.12 ± 0.85 ^b,c^ ^(1,2,7,8,10)^	<0.0001
P1 ^(1)^	zinc gluconate	5.79 ± 0.17 ^b,d^ ^(2,4,5,6,7,8,9)^	7.54 ± 0.38 ^a,c,d^ ^(0,3,7,9,10)^	5.86 ± 0.71 ^b,d (0,2,6,7,9,10)^	3.63 ± 0.56 ^a,b,c (0,3,4,6,7,9,10)^	<0.0001
P2 ^(2)^	zinc gluconate	9.90 ± 1.50 ^d (1,3,4,5,6,7,8,9,10)^	9.94 ± 1.67 ^d^ ^(0,3,7,9,10)^	8.01 ± 0.17 ^d (0,1,7,8,9,10)^	4.49 ± 0.66 ^a,b,c (0,3,7,9,10)^	<0.0001
P3 ^(3)^	zinc gluconate	5.40 ± 0.95 ^b,c,d (2,4,5,6,7,8,9)^	16.53 ± 0.62 ^a,c,d (1,2,4,5,6,7,8,9,10)^	7.76 ± 0.75 ^a,b (0,7,8,9,10)^	7.61 ± 0.53 ^a,b (1,2,7,8,10)^	<0.0001
P4 ^(4)^	zinc gluconate	13.92 ± 1.08 ^b,c,d (1,2,3,5,6,7,8,9,10)^	9.08 ± 0.65 ^a,c,d^ ^(0,3,7,9,10)^	7.23 ± 0.49 ^a,b (0,7,8,10)^	6.10 ± 0.34 ^a,b (1,7,8,9,10)^	<0.0001
P5 ^(5)^	zinc bisglycinate	19.35 ± 1.30 ^b,c,d (1,2,3,4,6,7,8,9,10)^	9.75 ± 0.63 ^a,c,d^ ^(0,3,7,9,10)^	7.22 ± 0.37 ^a,b (0,7,8,10)^	5.6 ± 0.13 ^a,b (7,9,10)^	<0.0001
P6 ^(6)^	zinc lactate	3.17 ± 0.46 ^b,c,d^ ^(1,2,3,4,5,7,10)^	6.83 ± 0.19 ^a,c^ ^(0,3,10)^	8.74 ± 1.34 ^a,b,d (0,1,7,8,9,10)^	6.53 ± 0.84 ^a,c (1,7,8,9,10)^	<0.0001
P7 ^(7)^	zinc picolinate	44.30 ± 1.35 ^b,c,d (1,2,3,4,5,6,8,9,10)^	36.09 ± 4.17 ^a (0,1,2,3,4,5,6,8,9,10)^	35.86 ± 1.9 ^a (0,1,2,3,4,5,6,8,9,10)^	35.94 ± 1.75 ^a (0,1,2,3,4,5,6,8,9,10)^	<0.0001
P8 ^(8)^	zinc citrate	2.24 ± 0.29 ^b,c,d^ ^(1,2,3,4,5,7,10)^	9.07 ± 0.59 ^a,c,d^ ^(0,3,7,9,10)^	5.02 ± 0.79 ^a,b,d (0,2,3,4,5,6,7,10)^	3.53 ± 0.46 ^a,b,c (0,3,4,6,7,9,10)^	<0.0001
P9 ^(9)^	zinc sulfate	2.00 ± 0.17 ^b,c,d^ ^(1,2,3,4,5,7,10)^	4.30 ± 0.88 ^a,d (0,2,3,4,5,7,8)^	5.30 ± 0.10 ^a,d (0,2,3,6,7,10)^	8.75 ± 1.69 ^a,b,c (1,2,4,5,6,7,8,10)^	<0.0001
P10 ^(10)^	zinc oxide	6.89 ± 0.84 ^b,c,d (2,4,5,6,7,8,9)^	1.77 ± 0.05 ^a (0,1,2,3,4,5,6,7,8,)^	1.05 ± 0.13 ^a (0,1,2,3,4,5,6,7,8,9)^	1.37 ± 0.06 ^a (0,1,2,3,4,5,6,7,8,9)^	<0.0001
ANOVA *p*	-	<0.0001	<0.0001	<0.0001	<0.0001	

X-Mean, SD—Standard Deviation. * letter or number markings should be compared considering symbols for mean values and symbols for diet types in the first row (the same letters) or dietary supplements in the first column (the same numbers).

**Table 5:** Bioaccessibility of Zn, considering the pharmaceutical form of the dietary supplements. The medians sharing the same letter in a row show a statistically significant difference between diets at *p* ≤ 0.0002. The medians sharing the same number in a column show a statistically significant difference between supplements at *p* ≤ 0.0002 *.

Pharmaceutical Formula	Without Diet ^(a)^ N Me (Q1–Q3)	Standard Diet ^(b)^ N Me (Q1–Q3)	Basic Diet ^(c)^ N Me (Q1–Q3)	High-Fiber Diet ^(d)^ N Me (Q1–Q3)	Kruskal–Wallis ANOVA *p*
Tablets ^(1)^	63 6.9 (3.52–12.71) ^(d)^	63 8.4 (6.95–9.74) ^(d) (2)^	63 6.8 (4.97–7.84) ^(d)^	63 4.25 (3.46–5.73) ^(a,b,c) (2,3)^	<0.0001
Coated tablets ^(2)^	9 4.8 (4.77–6.64) ^(b,c)^	9 16.5 (15.8–17.2) ^(a,c) (1)^	9 7.8 (6.84–8.49)	9 7.44 (7.13–8.26) ^(1)^	<0.0001
Capsules ^(3)^	18 22.41 (1.99–44.49)	18 17.88 (4.42–38.41)	18 19.79 (5.3–35.08)	18 22.5 (7.75–35.82) ^(1)^	0.8491
Kruskal–Wallis ANOVA *p*	0.516	<0.0001	0.0300	<0.0001	

Me—median, Q1—first quartile, Q3—third quartile. * letter or number markings should be compared considering symbols for mean/median values and symbols for diet types in the first row (the same letters) or dietary supplements in the first column (the same numbers).

## Data Availability

The original contributions presented in this study are included in the article/Supplementary Material. Further inquiries can be directed to the corresponding author.

## Supplementary Materials

The following supporting information can be downloaded at: https://www.mdpi.com/article/10.3390/nu18010094/s1, Table S1: Composition of diets used in the study; Table S2: Detailed composition of dietary supplements used in the experiment; Table S3: Operating parameters in the FAAS method; Figure S1: Bioaccessibility results with a detailed explanation of the interpretation of the provided letter and number symbols.
