# Supplementary material for: Influence of Diet on the Bioaccessibility of Zn from Dietary Supplements: Findings from an In Vitro Digestion Model and Analytical Determinations
Source: Nutrients. 2025 Dec 27;18(1):94. doi: 10.3390/nu18010094 (PMC12788010; doi:10.3390/nu18010094)
Supplement: Supplementary file 1 [file nutrients-18-00094-s001.zip › nutrients-4036765-supplementary-R.pdf]

**SUPPLEMENTARY FILE**

# Influence of Diet on the Bioaccessibility of Zn from Dietary Supplements: Findings from an In Vitro Digestion Model and Analytical Determinations

**Table S1. Composition of diets used in the study.**

| Type of the meal | Food product/meal | Amount [g/mL] |
|---|---|---|
| **Standard diet** | | |
| Breakfast | oat flakes | 60 |
| | milk UHT 3.2% | 250 |
| | raspberries | 100 |
| | banana | 60 |
| | coffee infusion | 200 |
| | sesame | 5 |
| | almods | 10 |
| Second breakfast | wheat bread | 50 |
| | cooked pork ham | 30 |
| | tomato | 130 |
| | butter | 10 |
| | apple | 100 |
| | orange juice | 200 |
| Lunch | tomato soup | 300 |
| | eggless pasta | 60 |
| | potatoes | 200 |
| | fired chicken breast | 150 |
| | cauliflower | 150 |
| | carrot | 45 |
| | leek | 15 |
| | mayonnaise | 10 |
| | salt | 2 |
| | apple compote | 200 |
| Dinner | omelet:<br>eggs<br>banana<br>oat flakes<br>olive oil | <br>100<br>50<br>30<br>5 |
| | plum powidl | 10 |
| | apricot | 100 |

| | | |
|---|---|---|
| | sugar | 5 |
| | black tea infusion | 250 |
| **Basic diet** | | |
| Breakfast | rye bread | 90 |
| | tomato | 120 |
| | turkey ham | 60 |
| | butter | 10 |
| | cucumber | 40 |
| | lettuce | 20 |
| | black tea infusion | 250 |
| Second breakfast | natural yogurt (2% fat) | 200 |
| | raspberries | 160 |
| | muesli with raisins and nuts | 100 |
| | coffee infusion | 250 |
| Lunch | breaded pork cutlet | 150 |
| | olive oil | 5 |
| | garlic | 5 |
| | potatoes | 190 |
| | green beans | 100 |
| | garden dill | 5 |
| | salt | 2 |
| | almonds | 10 |
| | mineral water | 200 |
| Dinner | Pancakes:<br>wheat flour<br>milk UHT 1.5%<br>eggs | <br>75<br>50<br>60 |
| | honey | 10 |
| | homogenized cottage cheese | 144 |
| | pear | 70 |
| | black tea infusion | 250 |
| **High-residue diet** | | |
| Breakfast | natural yogurt (2% fat) | 250 |
| | muesli with dried fruit | 100 |
| | wheat bran | 20 |
| | banana | 120 |
| | honey | 3 |
| | mineral water | 250 |
| Second breakfast | rye bread | 90 |

| | | |
|---|---|---|
| | tomato | 50 |
| | lettuce | 20 |
| | cucumber | 75 |
| | butter | 10 |
| | Mozzarella | 60 |
| | coffee infusion | 250 |
| Lunch | cooked pearl barley groats | 289 |
| | zucchini | 150 |
| | red pepper | 45 |
| | olive oil | 5 |
| | salt | 1 |
| | wheat bran | 20 |
| | leek | 20 |
| | roasted pork | 100 |
| | mineral water | 250 |
| Dinner | kefir (2% fat) | 330 |
| | wheat bran | 10 |
| | walnuts | 20 |
| | raspberries | 150 |
| | oat flakes | 50 |
| | black tea infusion | 250 |

‘

**Table S2.** Detailed composition of dietary supplements used in the experiment

| Product | Composition |
|---|---|
| **P1** | microcrystalline cellulose, zinc gluconate, magnesium stearate, copper gluconate |
| **P2** | glucose monohydrate, zinc gluconate, microcrystalline cellulose, magnesium salts of fatty acids, silicon dioxide |
| **P3** | cellulose, zinc gluconate, l-ascorbic acid, magnesium salts of fatty acids |
| **P4** | zinc gluconate, sorbitol, cellulose, magnesium salts of fatty acids |
| **P5** | Horsetail herb extract (Equisetum arvense L.), cellulose; vitamins [L-ascorbic acid – vitamin C, DL-alpha-tocopheryl acetate – vitamin E, nicotinamide – niacin, retinyl acetate – vitamin A, calcium D-pantothenate – pantothenic acid, |

| | |
|---|---|
| | cyanocobalamin – vitamin B12, cholecalciferol – vitamin D, pyridoxine hydrochloride – vitamin B6, riboflavin – vitamin B2, thiamine mononitrate – thiamine (vitamin B1), D-biotin – biotin, pteroylmonoglutamic acid – folic acid], nettle herb extract (Urtica dioica L.), zinc bisglycinate, cystine, coating [polyvinyl alcohol (PVA); titanium dioxide; polyethylene glycol, talc], bulking agent – cross-linked sodium carboxymethylcellulose; sodium selenate (IV), silicon dioxide, magnesium salts of fatty acids; copper bisglycinate |
| **P6** | microcrystalline cellulose, zinc lactate, magnesium salts of fatty acids, silicon dioxide. |
| **P7** | zinc (zinc picolinate), microcrystalline cellulose, capsule shell: gelatin, magnesium salts of fatty acids and silicon dioxide. |
| **P8** | microcrystalline cellulose, dicalcium phosphate; vitamin C (L-ascorbic acid); zinc (zinc citrate); stearic acid, hydroxypropyl methylcellulose, magnesium salts of fatty acids (magnesium stearate), silicon dioxide, polyethylene glycol |
| **P9** | Horsetail herb extract, beef gelatin, nettle herb extract, bamboo shoot extract, vitamin premix (L-ascorbic acid, nicotinamide, DL-alpha tocopheryl acetate, calcium D-pantothenate, riboflavin, pyridoxine hydrochloride, thiamine mononitrate, pteroylmonoglutamic acid, D-biotin, cyanocobalamin), mineral premix (iron lactate, zinc sulfate, manganese sulfate, copper (II) sulfate, potassium iodide, sodium selenite), L-methionine, pantothenic acid, para-aminobenzoic acid; silicon dioxide, magnesium salts of fatty acids, cellulose; D-biotin. |
| **P10** | Magnesium hydroxide, ginseng extract, sodium L-ascorbate, microcrystalline cellulose, cross-linked sodium carboxymethylcellulose, lecithin (soy), ferrous fumarate, maltodextrin, corn starch, glazing agents (magnesium salts of fatty |

| | |
|---|---|
| | acids, hydroxypropyl methylcellulose, shellac, carnauba wax), DL-alpha-tocopheryl acetate, nicotinamide, zinc oxide, calcium D-pantothenate, glycerol, copper (II) sulfate, iron oxides and hydroxides, pyridoxine hydrochloride, riboflavin, thiamine mononitrate, retinyl acetate, pteroylmonoglutamic acid, potassium iodide, sodium (IV) selenate, D-biotin, cholecalciferol, cyanocobalamin |

**Table S3. Operating parameters in the FAAS method.**

| | |
|---|---|
| Analytical line, reading time | Zn 213.9 nm, 4s |
| Flame type | air-acetylene |
| Background correction | deuterium |
| Singal type | continous |
| Fuel flow | 1.2 L/min |

**Figure S1. Bioaccessibility results with a detailed explanation of the interpretation of the provided letter and number symbols.**

| Dietary Supplement | Chemical Form | Without Diet $^{a}$ (n = 9) X±SD | Standard Diet $^{b}$ (n = 9) X±SD | Basic Diet $^{c}$ (n = 9) X±SD | High-fiber Diet $^{d}$ (n = 9) X±SD | ANOVA p |
|---|---|---|---|---|---|---|
| Without (0) | - | - | 19.43±0.72 $^{d}$<br>(1,2,4,5,6,7,8,9,10) | 16.18±0.20 $^{d}$<br>(1,2,3,4,5,6,7,8,9,10) | 8.12±0.85 $^{b,c}$<br>(1,2,7,8,10) | <0.0001 |
| P1 (1) | zinc gluconate | 5.79±0.17 $^{b,d}$<br>(2,4,5,6,7,8,9) | 7.54±0.38 $^{a,c,d}$<br>(0,3,7,9,10) | 5.86±0.71 $^{b,d}$<br>(0,2,6,7,9,10) | 3.63±0.56 $^{a,b,c}$<br>(0,3,4,6,7,9,10) | <0.0001 |
| P2 (2) | zinc gluconate | 9.90±1.50 $^{d}$<br>(1,3,4,5,6,7,8,9,10) | 9.94±1.67 $^{d}$<br>(0,3,7,9,10) | 8.01±0.17 $^{d}$<br>(0,1,7,8,9,10) | [illegible].49±0.66 $^{a,b,c}$<br>(0,3,7,9,10) | <0.0001 |
| P3 (3) | zinc gluconate | 5.40±0.95 $^{b,c,d}$<br>(2,4,5,6,7,8,9) | 16.53±0.62 $^{a,c,d}$<br>(1,2,4,5,6,7,8,9,10) | 7.76±0.75 $^{a,b}$<br>(0,7,8,9,10) | 7.61±0.53 $^{a,b}$<br>(1,2,7,8,10) | <0.0001 |
| P4 (4) | zinc gluconate | 13.92±1.08 $^{b,c,d}$<br>(1,2,3,5,6,7,8,9,10) | 9.08±0.65 $^{a,c,d}$<br>(0,3,7,9,10) | 7.23±0.49 $^{a,b}$<br>(0,7,8,10) | 6.10±0.34 $^{a,b}$<br>(1,,7,8,9,10) | <0.0001 |
| P5 (5) | zinc bisglycinate | 19.35±1.30 $^{b,c,d}$<br>(1,2,3,4,6,7,8,9,10) | 9.75±0.63 $^{a,c,d}$<br>(0,3,7,9,10) | 7.22±0.37 $^{a,b}$<br>(0,7,8,10) | 5.6±0.13 $^{a,b}$<br>(7,9,10) | <0.0001 |
| P6 (6) | zinc lactate | 3.17±0.46 $^{b,c,d}$<br>(1,2,3,4,5,7,10) | 6.83±0.19 $^{a,c}$<br>(0,3,10) | 8.74±1.34 $^{a,b,d}$<br>(0,1,7,8,9,10) | 6.53±0.84 $^{a,c}$<br>(1,7,8,9,10) | <0.0001 |
| P7 (7) | zinc picolinate | 44.30±1.35 $^{b,c,d}$<br>(1,2,3,4,5,6,8,9,10) | 36.09±4.17 $^{a}$<br>(0,1,2,3,4,5,6,8,9,10) | 35.86±1.9 $^{a}$<br>(0,1,2,3,4,5,6,8,9,10) | 35.94±1.75 $^{a}$<br>(0,1,2,3,4,5,6,8,9,10) | <0.0001 |
| P8 (8) | zinc citrate | 2.24±0.29 $^{b,c,d}$<br>(1,2,3,4,5,7,10) | 9.07±0.59 $^{a,c,d}$<br>(0,3,7,9,10) | 5.02±0.79 $^{a,b,d}$<br>(0,2,3,4,5,6,7,10) | 3.53±0.46 $^{a,b,c}$<br>(0,3,4,6,7,9,10) | <0.0001 |
| P9 (9) | zinc sulfate | 2.00±0.17 $^{b,c,d}$<br>(1,2,3,4,5,7,10) | 4.30±0.88 $^{a,d}$<br>(0,2,3,4,5,7,8) | 5.30±0.10 $^{a,d}$<br>(0,2,3,6,7,10) | 8.75±1.69 $^{a,b,c}$<br>(1,2,4,5,6,7,8,10) | <0.0001 |
| P10 (10) | zinc oxide | 6.89±0.84 $^{b,c,d}$<br>(2,4,5,6,7,8,9) | 1.77±0.05 $^{a}$<br>(0,1,2,3,4,5,6,7,8,) | 1.05±0.13 $^{a}$<br>(0,1,2,3,4,5,6,7,8,9) | 1.37±0.06 $^{a}$<br>(0,1,2,3,4,5,6,7,8,9) | <0.0001 |
| ANOVA p | - | <0.0001 | <0.0001 | <0.0001 | <0.0001 | |

X-Mean, SD – Standard Deviation

* letter or number markings should be compared considering symbols for mean values and symbols for diet types in the first row (the same letters) or dietary supplements in the first column (the same numbers)

| Dietary Supplement | Chemical Form | Without Diet $^{a}$ (n = 9) X±SD | Standard Diet $^{b}$ (n = 9) X±SD | Basic Diet $^{c}$ (n = 9) X±SD | High-fiber Diet $^{d}$ (n = 9) X±SD | ANOVA p |
|---|---|---|---|---|---|---|
| Without (0) | - | - | 19.43±0.72 $^{d}$<br>(1,2,4,5,6,7,8,9,10) | 16.18±0.20 $^{d}$<br>(1,2,3,4,5,6,7,8,9,10) | 8.12±0.85 $^{b,c}$<br>(1,2,7,8,10) | <0.0001 |

Interpretation: The bioaccessibility result for the standard diet differs significantly from the result for a high-fiber diet (p≤0.0002).

| Dietary Supplement | Chemical Form | Without Diet $^{a}$ (n = 9) X±SD |
|---|---|---|
| Without (0) | - | - |
| P1 (1) | zinc gluconate | 5.79±0.17 $^{b,d}$<br>(2,4,5,6,7,8,9) |
| P2 (2) | zinc gluconate | 9.90±1.50 $^{d}$<br>(1,3,4,5,6,7,8,9,10) |

Interpretation: The bioaccessibility result for the P1 supplement differs significantly from the result for the P2 supplement (p≤0.0002).
